# Patterns of Peripheral Nerve and Tendon Injury in Hand Trauma Patients in a Tertiary Care Hospital of Pakistan

**DOI:** 10.7759/cureus.12889

**Published:** 2021-01-24

**Authors:** Amna Ahmad, Shehzeen F Memon, Anosh Aslam Khan, Shahzeb A Memon, Sumeen Jalees, Sulhera Khan, Bareerah Shaukat

**Affiliations:** 1 Internal Medicine, Dow University of Health Sciences, Karachi, PAK; 2 Medical Education and Simulation, Dow University of Health Sciences, Karachi, PAK

**Keywords:** hand injury, peripheral nerve injury, combined nerve injury, tendon injury, combined tendon injury, trauma, poly digit trauma, karachi, pakistan, plastic surgery

## Abstract

Introduction

Traumatic injury to peripheral nerves is a major medical problem worldwide. Moreover, injury to the hand and wrist can lead to extreme morbidity and disable the injured for life. In this study, we highlight the most commonly damaged nerves and tendons that get ruptured in different types of hand trauma patients. No recent study has been done to document the etiologies and quantify the patterns of nerve and tendon involvement in hand injuries to the best of our knowledge.

Methodology

This was a cross-sectional study conducted at the largest trauma center in the city and the Plastic and Reconstructive Surgery ward of Civil Hospital, Karachi. A convenient sampling of 200 patients was done with the help of a preformed, well-structured questionnaire. Patients whose hand was injured solely were included in the study and those with the involvement of the whole limb or other parts of the body were excluded.

Results

We found that most males between the ages of 11 and 20 years presented predominantly with right-hand injury while working with machines. Nerve injury proved to be a rare occurrence. However, combined nerve injury of the ulnar, median, and radial nerve was seen in poly digit trauma. The median nerve was the most commonly damaged nerve in poly digit trauma. Among the tendon injuries, the incidence of combined tendon injury was the greatest. The flexor digitorum superficialis was the most common tendon injured overall.

Conclusion

This study significantly states that tendons are frequently injured in hand accidents. Plastic surgeons must also be aware of optimal nerve repair and reconstruction techniques to limit the physical disabilities and economic burden arising from nerve injury of the dominant hand.

## Introduction

Traumatic injury to peripheral nerves is a problem worldwide [1]. Direct injury to the hand and wrist can lead to extreme morbidity. Pakistan is an accident-prone nation where a large number of patients are admitted to emergency departments with trauma to various body parts, among which a majority suffer injuries to their hands. Hand injuries can disable patients for long periods or for life, considering that hand function is essential for almost all activities of daily living.

The most common causes of peripheral nerve injuries (PNIs) are mainly motor vehicle accidents, penetrating trauma after stabbing incidents and gunshots, and stretching or crushing after falls [2]. A study conducted at the Mayo Hospital in Lahore, Pakistan, in 2010, showed that the incidence of occupational hand trauma in patients who presented to one of the largest hospitals of Pakistan alone was 13.6% [3]. This did not include injuries sustained from road traffic accidents (RTAs), gunshots, fractures, and penetrating trauma. In recent years, no study has been done on the subject of documenting the etiologies and quantifying the patterns of nerve and tendon involvement in hand injuries, and those conducted elsewhere in the country have chiefly focused on management and post-operative results.

In this study, we highlight the most commonly damaged nerves and tendons that get ruptured in patients with different types of hand trauma presenting to a major trauma center in the largest metropolitan city of Pakistan, Karachi.

## Materials and methods

This is a cross-sectional study conducted at the Shaheed Mohtarma Benazir Bhutto Trauma Centre and the Department of Plastic and Reconstructive Surgery ward at Dr. Ruth K.M. Pfau Civil Hospital, Karachi, between the months of October 2019 and April 2020. A convenient non-probability purposive sampling of 200 patients was done using Open Epi version 3.03 at 95% confidence interval. A preformed questionnaire was sent to every consenting patient who presented to the trauma department and eventually the Plastic and Reconstructive Surgery ward with a hand injury. Only those patients were considered who presented exclusively with hand injuries. Patients with injuries not exclusively involving the hand met our exclusion criteria. Comatose and extremely traumatized patients who could not respond were also excluded. The questionnaire was composed of three parts. The first comprised questions on demographics such as the gender, age, and occupation of the patient. The second consisted of the nerves, namely, median, ulnar, and radial, arranged in a table for each digit separately. A neurological examination was done to assess the nerves affected. Third, the tendons involved were marked against each digit in a tabulated form. Data were entered in Statistical Package for the Social Sciences version 22 (IBM, Armonk, NY, USA) and analyzed with statistical tests to find out the frequencies of categorical variables like age, gender, cause of injury, location of the injury, and hand dominance. The Chi-square test was applied thrice for finding out the relationship between the tendon injured and the digit involved, the nerve injury and the digit involved, and, lastly, between the area of injury and the nerve that was injured. A p-value was calculated to observe the statistical significance of the areas being explored in the study. It was found to be significant (p-value < 0.05) in the former two cases and insignificant (p-value > 0.05) in the last.

## Results

The study showed that most patients who came with hand injuries were between the ages of 11 and 20 years (32.5%) as seen in Table 1. Out of 200 cases, 131 presented predominantly with right-hand injury, among which 67 had no nerve involvement at all. This is elaborated in Table 2.

**Table 1:** Frequency of patients in terms of their ages.

Age of the patients (years)	Number of patients N (%)
0-10	24 (12.0%)
11-20	65 (32.5%)
21-30	64 (32.0%)
31-40	31 (15.5%)
>40	16 (8.0%)

**Table 2:** Frequency of nerve involvement in the right and left hand.

Nerve involved	Injured hand (N)	Injured hand (N)
	Right hand	Left hand
No nerve involved	67	37
Median	16	10
Ulnar	19	12
Radial	5	4
Combined	24	6
	Total = 131	Total = 69

As seen in Table 3, most of the patients (N = 90) received their injuries at their workplace followed by injuries at residence (N = 54).

**Table 3:** Frequency of patients in terms of location of injury.

Location of injury	Number of patients (N)
Home	54
Work	90
Roads	44
Sports	3
Others	9

A majority of the patients faced accidental injuries, with the most common etiology being working with machines (N = 74) followed by glass cuts (N = 50) as the second common, as indicated by Table 4.

**Table 4:** Frequency of patients in terms of causes of injury.

Cause of injury	Number of patients (N)
Knife	12
Glass	50
Machinery	74
Burns	7
Explosives	3
Rollers	12
Others	42

A great proportion of the patients did not have any nerve injury at all, and if they did have any, more than one nerve was involved in the combination. Tendons were seen to be ruptured very frequently, with the rupture of flexor digitorum superficialis (FDS) being a regular recurrence while a mixture of tendon involvement was seen among other cases.

**Figure 1:**
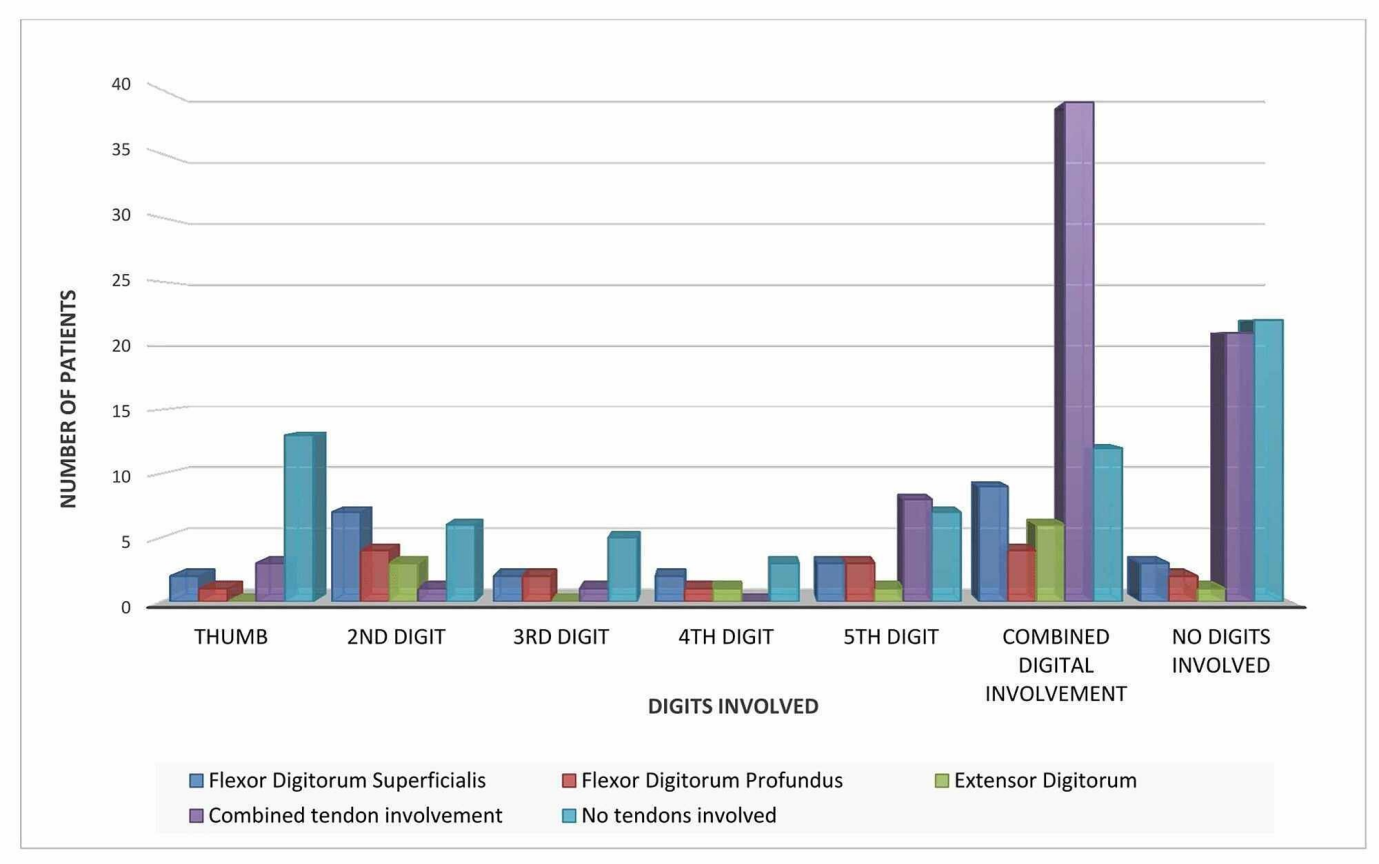
Tendon injury in relation to digits Involved.

Among the tendon injuries, the incidence of combined tendon injury was the greatest. Individually, none of the digits suffered greatly but there was a predominance of combined tendon damage leading to poly digit trauma (39 cases). Combined tendon damage also occurred without a visible skin injury to the digits in 21 cases. The FDS was the most common tendon injured overall (28 cases) (Figure 1). The p-value for the relationship between tendon injury and the digits involved was 0.001 which was statistically significant.

**Figure 2:**
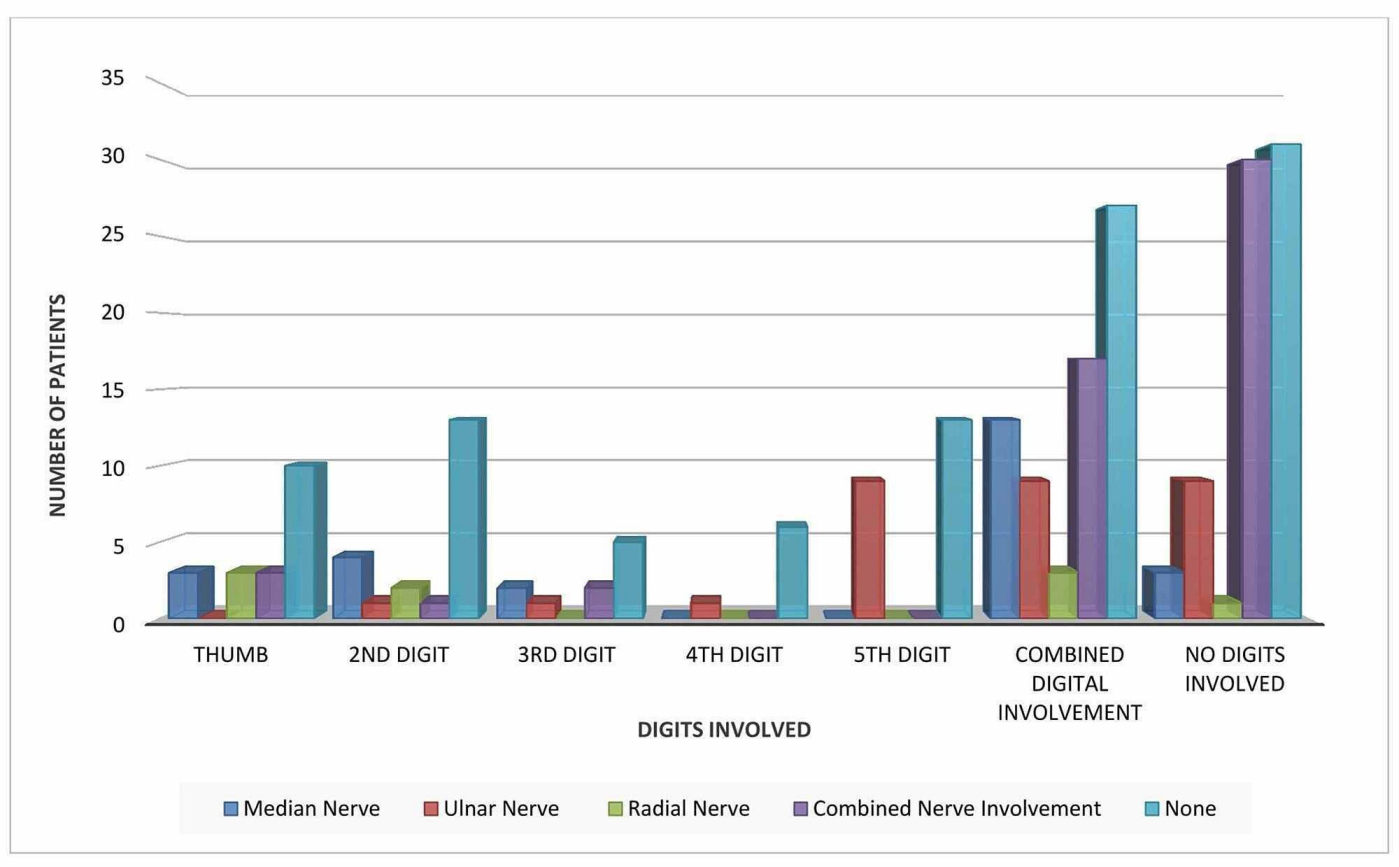
Nerve injury in relation to the digits involved.

Nerve injury proved to be a rare occurrence. However, combined nerve injury of the ulnar, median, and radial nerve was seen in poly digit trauma. The median nerve was the most commonly damaged nerve solely in poly digit trauma (13 cases) while 30 cases had internal combined nerve involvement without visible trauma to the digits (Figure 2). The p-value was statistically significant at 0.009.

**Figure 3:**
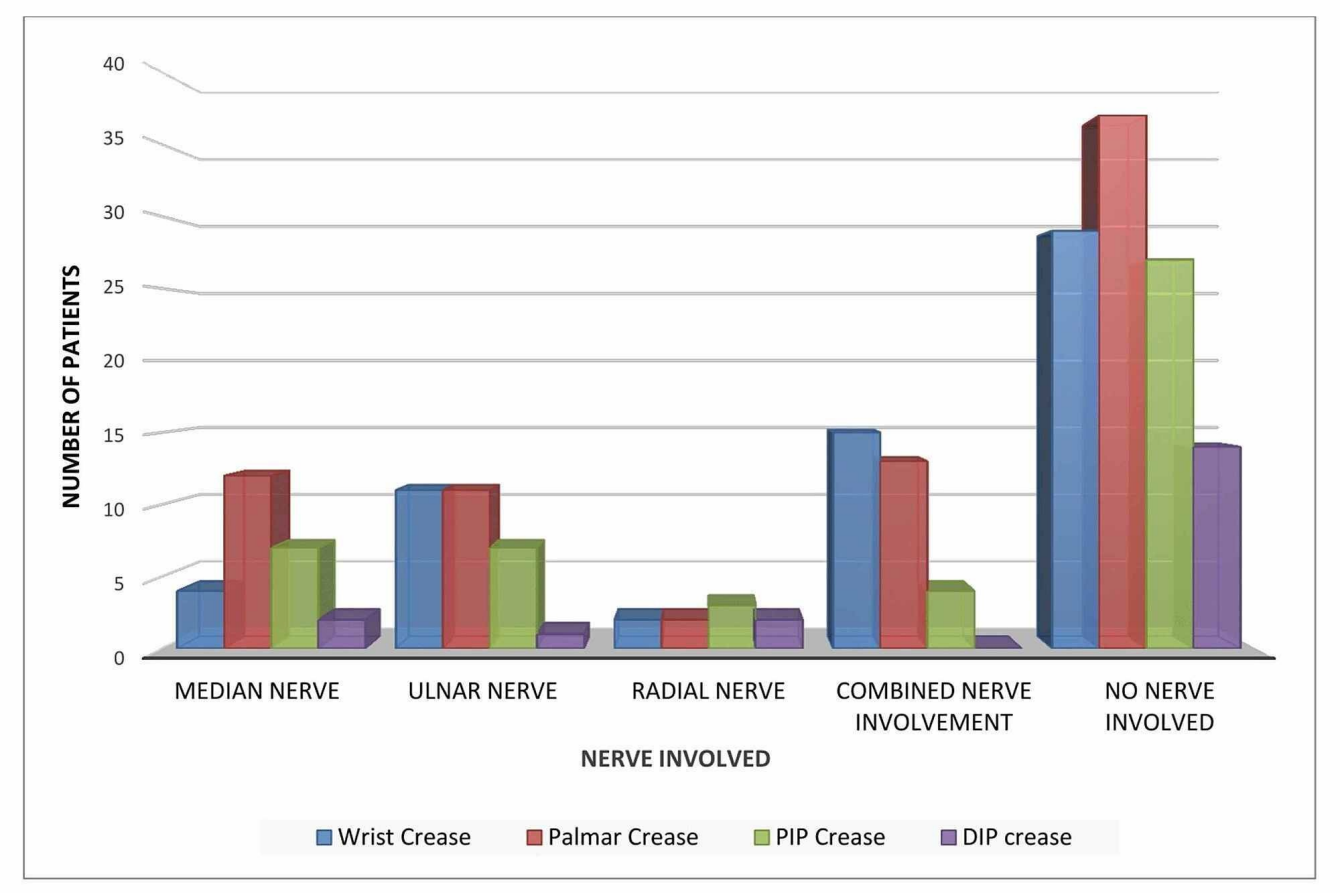
Nerve injury in relation to the extent of hand injury.

In relation to the area till which nerve injury occurred, injuries that spread till the palmar crease had no nerve involvement in 37 cases. Although a pattern of combined nerve injury was seen till the palmar crease, the median nerve was found to be the most damaged (12 cases). Injuries sustained till the wrist crease majorly had no nerve damage (29 cases) but did have combined nerve trauma in 15 cases. Among the injuries that spread to the proximal interphalangeal (PIP) crease, most (27 cases) did not have nerve damage. The injury that spread till the distal interphalangeal crease was the rarest and did not have any nerve damage associated with it (Figure 3). The p-value for this relationship was non-significant at 0.182.

## Discussion

The mean age for PNI was between 20 and 30 years in various previous studies [4-6]. In contrast, our study found that the prevalence of hand trauma was greatest in the 11-20 years age group, portending long-standing disability from an earlier age in patients with unfavorable outcomes. Such disability at a young age is a national socioeconomic burden as individuals in this age group constitute or will soon constitute a significant proportion of the workforce. The predominance of hand injury in our study was greater in males as in other studies analyzing gender distribution in trauma victims [4,7]. A study done in India with workplace setups similar to ours reported that most nerve injuries occurred at the workplace of the victim [5]. The most common cause of nerve injury in our study are injuries sustained via machinery and glass at the workplace, suggesting that proper safety measures must be taken in industries to reduce the burden of occupational hand trauma.

A regional audit of 4,873 hand and wrist injuries by Hill et al. reported the frequency of trauma in both the right hand (51.8%) and the left hand (45.4%) to be almost equal, and a study done in 2009 reported peripheral nerve involvement to be slightly greater on the right side of the body, although neither of these studies was restricted only to peripheral nerve involvement of the hand [1,4]. Nerve injury appeared to be greater in the right hand according to our study probably because of right-hand dominance.

Noble et al. found that the ulnar nerve was the nerve most commonly injured in upper extremity trauma similar to our study, which found that the ulnar nerve overall was largely involved in most cases of hand trauma [8]. In cases of poly digit trauma, the median nerve was the most common nerve affected. Injuries to ulnar and median nerves usually result in immobilization and non-usage of the hand for a long span of time which is again concerning as the most frequently affected age group in our study were young men [9].

A study conducted by Ahmad et al. in Pakistan analyzing flexor tendon injuries demonstrated that the FDS and flexor digitorum profundus were the most common tendons involved, as in the case of our study [10]. The middle and ring fingers sustained the most tendon injuries in their study, while the fifth finger had the most tendon injuries in our study. A retrospective study conducted by Kotwal et al. demonstrated that 63% of patients with a nerve injury also had coexisting tendon injuries; hence, the possibility of an associated tendon injury in a patient presenting with a nerve injury should not be ignored [11]. Pakistan is a low-resource, third-world country where the cost of treatment of patients with nerve and tendon injuries in tertiary care public hospitals places an excessive financial burden on the state. The study by Hill et al. likewise stated that concomitant tendon and nerve injury tend to double treatment expenses [4].

Trauma to the underlying nerves and tendons without any associated visible digital trauma underscores the fact that nerve and tendon damage may not be immediately visible, delaying the diagnosis. Therefore, in certain cases, the need for surgical exploration cannot be undermined.

No recent literature exists on peripheral nerve trauma in the hand from Pakistan; therefore, no local comparisons with this study can be made. There is a great need for further research to document the etiology and patterns of nerve involvement in trauma to the hand in our region. It is alarming that the majority of victims who presented with occupational hand trauma were children in the age group of 11-20 years. This hints towards the probability of prevalence of child labor in Pakistan that needs to be investigated and stopped so as to prevent our young generation from becoming not only victims of child labor but also the lifelong disability that it may give rise to.

## Conclusions

This study is the first of its kind in Karachi to analyze the patterns of peripheral nerve involvement in the hand, significantly showing significant involvement of tendon injury in hand accidents. As hand trauma takes up a major chunk of surgeries each day, plastic surgeons must be aware of optimal nerve repair and reconstruction techniques to limit the physical disability and economic burden arising from nerve injury of the dominant hand.
